# Revisiting the learning curve (once again)

**DOI:** 10.3389/fpsyg.2013.00982

**Published:** 2013-12-26

**Authors:** Steven Glautier

**Affiliations:** School of Psychology, University of SouthamptonSouthampton, UK

**Keywords:** learning curve, averaging, individual differences, mathematical model, environment structure

## Abstract

The vast majority of published work in the field of associative learning seeks to test the adequacy of various theoretical accounts of the learning process using average data. Of course, averaging hides important information, but individual departures from the average are usually designated “error” and largely ignored. However, from the perspective of an individual differences approach, this error is the data of interest; and when associative models are applied to individual learning curves the error is substantial. To some extent individual differences can be reasonably understood in terms of parametric variations of the underlying model. Unfortunately, in many cases, the data cannot be accomodated in this way and the applicability of the underlying model can be called into question. Indeed several authors have proposed alternatives to associative models because of the poor fits between data and associative model. In the current paper a novel associative approach to the analysis of individual learning curves is presented. The Memory Environment Cue Array Model (MECAM) is described and applied to two human predictive learning datasets. The MECAM is predicated on the assumption that participants do not parse the trial sequences to which they are exposed into independent episodes as is often assumed when learning curves are modeled. Instead, the MECAM assumes that learning and responding on a trial may also be influenced by the events of the previous trial. Incorporating non-local information the MECAM produced better approximations to individual learning curves than did the Rescorla–Wagner Model (RWM) suggesting that further exploration of the approach is warranted.

Objectively, associative learning theory is a thriving enterprize with a rich tradition of experimental work interpreted through the lenses of sophisticated mathematical models. However, there remains a fundamental empirical observation that is still not well captured by these models. Despite many attempts to provide an adequate account of the learning curves that are produced, even in a simple conditioning experiments, there is still considerable unexplained variation in these curves. For example, many formal models of learning lead us to expect smooth learning curves but these are seldom observed except at the level of average data. Small departures from a theoretical curve can be tolerated as measurement error but when this error is large the model must be called into question and some authors have concluded that associative models are fundamentally wrong. An alternative position, the one adopted in the current paper, is that the associative framework is essentially correct. However, it is argued that much more accurate modeling of individual learning curves is needed and can be achieved by using a more detailed representation of the stimuli provided by the learning environment. In what follows I will describe the application of a mainstream model of associative learning, the Rescorla–Wagner Model (RWM, Rescorla and Wagner, 1972), to individual learning curves. Best fitting RWM learning curves will be compared to best fitting learning curves from a modified approach which uses a more detailed representation of the stimulus environment. The modified approach, which I have named the Memory Environment Cue Array Model (MECAM), works algorithmically in the same way as the RWM but additionally incorporates memory buffers to hold representations of the previous trial's events. These memory representations are then processed alongside representations of the current trial. The question addressed in this paper is whether or not we can improve on the standard RWM to obtain a better model for individual learning curves by using the MECAM's extended description of the stimulus environment. Before describing the details of the MECA Model, a brief overview of the RWM and learning curve problems will be presented as a background.

The RWM is widely regarded as a highly successful and relatively simple model of associative learning (c.f. Miller et al., 1995, for an overview). In the RWM learning is described in terms of the growth of associative strength between mental representations of stimulus events. The RWM was originally developed to describe animal learning experiments, in particular experiments using Pavlovian conditioning procedures. During Pavlovian learning the RWM assumes associations are developed between mental representations of the conditioned stimulus (CS) and the unconditioned stimulus (US). For example, the experimenter may present a tone (CS) and a few seconds later an electric shock (US). After a number of CS–US pairings the experimental animal exhibits conditioned responses (CRs e.g., freezing) when the CS is presented and this is said to occur because the associations between the CS and the US representations allow excitation to spread from one representation to the other. Thus, presenting the CS excites the representation of the US and produces the observed CRs. Informally, the presence of the CS generates an expectancy of the US. The RWM principles are sufficiently general to have been successfully imported into new domains. Since its development as a model of Pavlovian conditioning in animals the RWM has been considered a viable candidate model in a variety of human learning tasks including predictive, causal, and Pavlovian learning (e.g., Dickinson et al., 1984; Lachnit, 1988; Chapman and Robbins, 1990).

(1) ΔV=αβ(λ−ΣV)

Equation (1) is the fundamental RWM learning equation. In the equation Δ*V* is the change in the associative strength between the mental representation of a predictive stimulus (such as a tone CS) and the representation of the outcome (such as a shock US) that occurs on a single learning trial. Δ*V* is a function of two learning rate parameters, α for the CS and β for the US, and the parenthesized error term. In the error term λ is the value of the US on that trial (usually 1 or 0 for the occurrence and non-occurrence of the US, respectively) and Σ*V* is the summed associative strength of all the predictors that are present on the trial. The RWM is said to be error driven and competitive. It is error drive in the sense that the amount of learning depends on the difference between what occurs, λ, and what was expected, Σ*V*. It is competitive in the sense that the updates applied to the associative strength of a stimulus depend not just on the strength of that stimulus but also on the strength of all the other stimuli that are present on the trial—Σ*V* is used in the error term rather than *V* alone. This competitive error driven formulation is a defining feature of the RWM and has been adopted in many neural network models of learning (c.f. Sutton and Barto, 1981).

Historically, analysis of learning curves has been an important testing ground for theories of learning. Any credible theory of learning must be able to account for state transitions, as well as steady state performance. Each theory of learning makes characteristic predictions for the shape of the learning curve, the RWM is no exception. Referring to Equation (1) we can see that associative strength increases as a fixed proportion (αβ) of the difference between the current associative strength and the asymptote. From the RWM we therefore expect orderly negatively accelerated learning curves. Because each theory of learning makes characteristic learning curve predictions, in principle, analysis of learning curves should be theoretically decisive. Unfortunately, the utility of this approach has not been realized because of the empirically observed heterogeneity in learning curves. Smooth monotonic, S-shaped, and stepped curves have all been seen at one time or another leading Mazur and Hastie to comment “In fact, learning curves of almost every conceivable shape have been found.” (Mazur and Hastie, 1978, p. 1258). No doubt some of this variability can be accounted for by the type of task. For example, many tasks have several components, some of which might be relatively easy to learn. On this basis a task composed of simple and difficult components could produce rapid improvements in performance in the first few trials after which the rate of improvement would decline. On the other hand, a multicomponent task which involved several equally difficult components could produce a less variable rate of improvement. Thus, the shape of the learning curve might be affected by the structure of the task that is presented and may not be straightforwardly diagnostic of the underlying process. Nevertheless, despite these interpretational problems, analyses of learning curves led to a widespread acceptance of the principle embodied in Newell and Rosenbloom's Power Law of learning (Newell and Rosenbloom, 1981). The Power Law of learning is based on an equation of the form *P* (Correct Response) = 1−α*t*^−β^ where *t* is the trial number in the series, α and β are parameters of the curve. An equation of this type generates a curve in which the proportional progress toward asymptote declines with trials. In contrast, an exponential function *P* (Correct Response) = 1 − α*e*^−α*t*^ generates a curve in which the proportional progress toward asymptote remains constant with trials. Although there is now doubt about the status of the Power Law (Heathcote et al., 2000; Myung et al., 2000), the point to draw attention to is the critical theoretical position that has been occupied by learning curve analyses and the fact that this theoretical promise has not been realized—we cannot confidently rule in or out the RWM on the basis of its characteristic exponential form.

However, when individual learning curves are considered it is not surprising that it has proved difficult to clearly determine whether learning curves are best characterized by power functions or by exponential functions. These are relatively subtle differences occurring against a background of great variability from one participant to the next. At the level of individual learning curves there is actually little evidence of smooth learning functions, let alone clearly distinguishable exponential or power functions. One solution to this problem has been to average the individual data and then try to find the function which best describes the average curve. These average data can be well approximated by exponential or power functions. Unfortunately, this is not a viable solution because averaging of the data points generated by a function does not, in general, equal the application of that function to the average i.e., Mean(*f*(*i*), *f*(*j*), … *f*(*n*)) ≠ *f*(Mean(*i*, *j*, … *n*)) (Sidman, 1952; Estes, 2002).

Although it has not been possible to adjudicate between exponential and power models of learning, analysis of the learning curve continues to stimulate important theoretical debates. The difficulty with trying to represent individual learning curves with the orderly incremental learning functions used in associative models of learning such as the RWM has led some authors to question the applicability of associative models, as a class, and to propose alternative, non-associative, mechanisms for learning. Köhler's (1925) work on insight learning is an early example, more recent statements come from Nosofsky et al. (1994) and Gallistel and Gibbon (2002). Nosofsky et al. described the Rule-Plus-Exception (RULEX) model of classification learning in which learning is conceived of as the acquisition of simple rules for classification e.g., “if feature A is present the item belongs to category X.” In RULEX simple rules are tried first and, if these fail, exceptions and more complex rules may then be tried. The relevance of RULEX in the current context is its supposition that individual learners will test and adopt rules in idiosyncratic ways and that acquisition of a successful rule will result in step changes in learning performance. Therefore individual curves will be characterized by abrupt changes and the location of these changes in a sequence of learning will vary randomly from participant to participant. Gallistel and Gibbon (2002) advocate an information processing model in which a response is generated when the value of a decision variable reaches a threshold value. Individuals vary in terms of the threshold value and in terms of the value of the decision variable. The result is that learning curves are expected to contain step changes varying in location from individual to individual (Gallistel et al., 2004). Neither of these models anticipate smooth individual learning curves but in both cases averaging of the individual curves produced by the models would result in smoothing. In both cases non-associative cognitive processes are proposed to explain the patterns observed in the individual data.

It is accepted that the RWM, and other modern associative models, only provide poor approximations to individual learning curves. Individual curves are highly variable from participant to participant. For example, looking ahead to the dataset to be described in more detail below, it can be seen that some participants learn quickly, apparently hitting upon a solution straight away (e.g., Figure 7 middle panel, square symbols). Some learn quickly but might take several trials to find the solution (e.g., Figure 7 left middle panel, square symbols). Others learn slowly with responses gradually approaching an asymptote as might be expected from the RWM (e.g., Figure 4 left middle panel, square symbols). Furthermore, responses are often unstable showing trial-to-trial fluctuations (e.g., Figure 2 left top panel, square symbols). Instability can occur even if an asymptote appears to have been reached (e.g., Figure 5 right middle panel, square symbols). In these respects this human predictive learning data contains the same features described by Gallistel et al. (2004) in a variety of animal learning tasks including autoshaped pigeon key presses and eye-blink conditioning in rabbits.

The main purpose of the current paper is to explore a development in the application of the RWM with the aim of trying to obtain a better approximation to individual acquisition data within a simple associative framework. Readers familiar with associative approaches related to Stimulus Sampling Theory (Estes, 1950; Atkinson and Estes, 1963) may question the appropriateness of the RWM as the origin for this endeavor when two basic principles of Stimulus Sampling Theory appear to provide an initial step in the right direction. These principles are those of probabilistic environmental sampling and all-or-none learning (see also original paper and recent review of all-or-none learning debate Rock, 1957; Roediger and Arnold, 2012). In Stimulus Sampling Theory it is assumed that each learning trial involves a probabilistically obtained sample of stimulus elements. Given that the sampled elements may be connected to different responses there is a built in mechanism that can produce trial-by-trial response variability. Furthermore, because associations are assumed to be made in an all-or-none fashion when reinforcement occurs step-wise changes in behavior are expected. However, although Stimulus Sampling Theory is prima-facia a strong candidate with which to tackle the characterization of individual learning curves the RWM was chosen as a basis because of its competitive error driven formulation which has proven to be extremely useful (but not universally successful c.f. Miller et al., 1995) in accounting for a wide variety of other learning phenomena.

In developing the framework provided by the RWM the starting point was to question the assumption that participants in a learning experiment base their expectations and learning for the current trial just on the stimuli present on that trial. Actually, the learning trial is an artificial structuring of events created largely for the convenience of the experiment and there is no good reason to believe that participants actually parse their experience in this way. In fact most learning experiments have short inter-trial-intervals of just a few seconds (e.g., in Thorwart et al., 2010, ITIs of 4 s and 6 s were used in two different experiments) so that participants will still have fresh in their minds a memory of the previous trial. Evidence from several sources confirms that participants do remember previous trials and these memories can influence behavior on the current trial. For example participants remember when they have had a series of reinforced or non-reinforced trials and this affects what they expect to happen on the current trial (Perruchet et al., 2006). In the Perruchet task a long sequence of non-reinforced trials leads to an expectation that the next trial will be reinforced and vice-versa. Participants also respond to trial sequence information so that reaction time is reduced if the sequence is predictive of the response requirement, and this can occur without the participants developing a conscious expectancy for the outcome (e.g., Jones and McLaren, 2009).

In the MECA Model it is proposed that remembered stimulus elements from the previous trial are processed along with current elements and can therefore acquire associations with the outcome and contribute to the control of expectations in the same way as current elements. The MECAM works by utilizing three memory buffers in which representations of the current trial are stored alongside representations of the previous trial. The MECAM encodes the stimuli of the current trial in the *primary buffer*. Experimenter defined stimulus elements serving the CS roles are encoded along with unique configural cues (Rescorla, 1973) representing pairwise interactions between experimenter defined stimulus elements. The *secondary buffer* is a copy of the primary buffer from the previous trial plus a representation of the outcome event that served as the US on the previous trial. The *interaction buffer* contains pairwise configural cue representations for the elements from the current and previous trial. The MECAM contains and parameter ω which weights the secondary and interaction buffers. Setting these weighting parameters to zero reduces the MECAM to the RWM. The Appendix contains a detailed description of the implementations of the RWM and MECAM that were used in the simulations that will be reported below.

(2) ΔV=ωαβ(λ−ΣV)

Equation (2) provides the learning equation used in MECAM. There is no difference between the RW and MECA models in the way associative strength updates are made except that in the MECAM the additional parameter ω is combined multiplicatively with the learning rate parameters α and β (compare Equation (1) and Equation (2)). The value of ω is allowed to vary for each cue according to the buffer in which the cue is defined. Primary buffer cues have ω = 1 whereas for secondary and interaction buffers 0 ≤ ω ≤ 1. Further details are provided in the Appendix and below there follows a short outline of MECAM's operation.

Table A2 provides an illustration of the operation of MECAM's buffers during three conditioning trials. On the first trial experimenter defined cues A and B are present along with the US outcome (an AB+ trial). The cue elements A and B appear in the primary buffer as does the configural cue ab. Cue ab is a theoretical entity used to represents the conjunction of the elements A and B. Because this is the first trial the secondary and interaction buffers are empty and only the cues A, B, and ab will have their associative strengths updated. At this point the RWM and MECAM are entirely equivalent. Differences appear on the second trial because now MECAM processes memorial representations of the events of the first trial alongside the events that occur on trial two. On trial two, three cues A, B, and C are present and there is no outcome (an ABC- trial). Configural cues ab, ac, and bc are used to represent the pairwise conjunctions of the cue elements. Thus, on trial two, there are six stimuli present in the primary buffer. There is no difference between the RWM and the MECAM in the processing of primary buffer cues. However, the MECAM additionally operates on the cue representations which now occupy the secondary and interaction buffers. There are two aspects of this operation. First, the existing associative strengths of the secondary and interaction buffer cues are combined with those in the primary buffer to produce Σ*V*. In this way the contents of all three buffers contribute to the outcome expectation for the trial. Second, the associative strengths of the cues present in all three buffers are updated. The cues present in the primary buffer are always just those that occur on the current trial (including configural components) whereas the secondary buffer contains a copy of all of the stimuli that occurred on the previous trial. These remembered stimuli have their own representations and associative strength. Thus, stimuli A and A_*t* − 1_ are distinct entities, as are ab and a_*t* − 1_b_*t* − 1_. Because the outcome of the previous trial is just as likely, if not more likely, to be remembered than the cues, the previous trial outcome is also coded as one of the remembered stimuli in the secondary buffer (O_*t* − 1_). The interaction buffer encodes a subset of the configural cues that are processed by MECAM. This subset consists of pairwise configurations of the elements of the current trial and the remembered elements from the previous trial. In the Trial 2 example shown in Table A2 the elements are A, B, and C from the current trial and elements A_*t* − 1_, B_*t* − 1_, and O_*t* − 1_ from the previous trial. This results in nine configural cues appearing in the interaction buffer. The use of three buffers allows different ω weights to be used for different classes of stimulus entity. The third trial illustrated in Table A2 gives a further example of how the buffer states change on the next, BC−, trial.

The MECAM is predicated on the assumption that the source of the behavioral complexity in individual learning curves is to be found in the environment to which the participants are actually exposed. A corollary is that even if the RWM is correct in its basic principles then simulations of individual participant behavior using the RWM will be inaccurate unless the input representations for the simulation match those in the individual's learning experience. The MECAM hypothesis is that during learning some of the influences on participant responding will be due to learning of associations between trial outcomes and memories of events occurring on previous trials. If this is correct then MECAM simulations, which incorporate representations of the previous trial events as inputs to the learning and expectations for the current trial, would provide better approximations to individual learning curves than the RWM, which involves learning and expectations only for current trial events. The experiments reported below involved participants making judgements about the likelihood of an outcome in each of a series of trials. Participant responses were in the form of ratings on an 11-point scale, running from 0—event will not occur, through 5—event will/will not occur with equal likelihood, to 10—event will occur. However, these judgements are not represented directly in either the RWM or MECAM. The currency of these models is the unobserved theoretical quantity of “associative strength.” Therefore, to model the changes in these judgements during learning it was necessary to find an appropriate way to map between the theoretical quantity of associative strength and observed judgements.

Unfortunately there is little agreement on the specific mapping between association strength and behavioral response (Rescorla, 2001). This situation may seem to be a fatal flaw in any attempt to provide a testable associative theory but the problem can be circumvented in some cases by making the minimal assumption of a monotonic relationship between the strength of the CRs and association strength. This is reasonable when there are qualitatively different predictions for the effect of an experimental manipulation for the theories under consideration. For example, in a feature-negative experiment one stimulus is reinforced (A+ trials) but a compound stimulus is non-reinforced (AB− trials). The effects of adding a common feature to these trials, to give AC+ and ABC− trials, differs qualitatively for leading associative models (Thorwart et al., 2010). According to the RWM the common-cue manipulation should make the discrimination between reinforced and non-reinforced trials easier whereas according to an alternative associative model the discrimination should become more difficult (Pearce, 1994). Thus, that comparison (Thorwart et al., 2010) between two associative models only required the assumption of a monotonic mapping between association strength and response strength. However, in the current work there are no experimental manipulations with qualitatively different predictions for the RWM and MECAM. Instead, a quantitative comparison of the goodness of fit between RWM and MECAM predictions and participant responses was carried out. This needs a mapping between the model currency of association strength and behavioral response and a choice of mappings is available. Two mapping functions were selected and compared. It was assumed that strength of association could be treated as type of stimulus to which participants would respond when asked to make their predictive judgements so that a psychophysical scaling would be appropriate. Two psychophysical functions have frequently been used to relate stimulus magnitude to perceived stimulus intensity, one based on Stevens' Power Law the other based on Fechner's Law (e.g., Krueger, 1989). In the analyses below simulations were carried out using both of these mappings and comparisons between them were made.

## Methods

The simulations reported below used data from a series of six different multi-stage experiments. These experiments all used a computer-based predictive learning task with a first stage consisting of AX+, AY+, BX−, and BY− trials. Data from these trials was used in the following analyses. In this notation the letters indicate which cues are present on a trial, the plus and minus signs indicate the presence or absence of the outcome. Analysis 1 used data from Experiment 1. The data from experiments 2–6 were combined and treated as data from a single experiment, hereinafter referred to as Experiment 2, in Analysis 2.

### Experimental method

The computer-based predictive learning task was presented as a simple card game in which the participants had to learn which cards would be winning cards. Participants were presented with a series of trials each beginning with a display of a card. Participants then used the keyboard cursor keys to adjust an onscreen indicator to indicate their judgement of the likelihood that the card would win. After the participant made a judgement the trial ended with feedback on whether the card won or lost. The cards had distinctive symbols and background colors such that the symbols and colors could be used as cues to distinguish the winning and losing cards. Experiment 1 and Experiment 2 used different computer programs for implementation of the task, had different numbers of trials in the learning sequence, and used different participant populations. The five experiments that were combined for Experiment 2 were the same on all of these variables so they were analyzed together as a single experiment. Replication of the analyses on the datasets of Experiment 1 and Experiment 2 provided a test of reliability and generality of findings.

#### Participants

Sixty-one participants took part in Experiment 1. Their average age was 17 years and they included 18 males. They were recruited during a site visit to a sixth form (age 16–18) college in Hampshire, UK. Participation was voluntary. One hundred and forty-four participants took part in Experiment 2. Their average age was 22 years and they included 41 males. They were recruited from the student and staff at the University of Wales Swansea campus and were paid £3 for participating.

#### Apparatus

In Experiment 1 participants were tested in groups at three computer workstations housed in a mobile research laboratory set up in the load compartment of a specially equipped Citroen Relay van. To minimize interference between participants auditory stimuli were presented over headphones and seating was arranged so that participants could easily view only their own computer screen. The screens measured 41 cm × 26 cm (W × H) and were run in 32 bit color mode with pixel resolutions of 1440 × 900. The display was controlled by a computer program written in Microsoft Visual Studio 2008 C# language and used XNA Game Studio Version 3.1 for 3D rendering of the experimental scenario. In Experiment 2 participants were tested individually in small experimental cubicles with sounds presented over the computer speakers. The screens measured 28 cm × 21 cm (W × H) and were run in 8 bit color mode with pixel resolutions of 640 × 480. The display was controlled by a computer program written in Borland Turbo Pascal.

#### Design and procedure

In all experiments participants were given a brief verbal description of the procedure before reading and signing a consent form. Next, a more detailed description of the procedure was presented on-screen for participants to read. In Experiment 1 the on-screen information was given along with a voiceover of the text, played through the headphones. The text from Experiment 1 is reproduced in full below. The text used in Experiment 2 had minor wording differences but conveyed the same information.

Thank you for agreeing to take part in this experiment. During the experiment you will be shown a series of “playing cards” on the computer screen. The cards were played in a game at Poker Faced Joe's Casino. The experiment is divided into a series of trials, each trial representing one card game. On each trial you have to rate the likelihood that the cards on the screen will WIN or LOSE. Make your rating by adjusting the indicator using the UP and DOWN arrow keys. When you have made your rating press RETURN. When you press return the cards will be turned over and you will find out whether they win or lose. Your job is to learn what outcome to expect. At first you will not know what to expect so you will have to guess. However, as you learn, you should aim to make your predictions as accurate as possible, to reflect the true value of the cards that are in play. Review these instructions on the screen. When you are sure that you understand what is required, press the key C to continue. Please note, Poker Faced Joe's is an imaginary casino you will not lose or gain any money by the rating you make. However, please try to make your judgements as quickly and as accurately as you can. Ask the experimenter if you have any questions or press the key C to begin.

After reading the instructions participants initiated the experimental trials with a key press. There then followed a series of trials. Each trial was one of four types; AX+, AY+, BX−, or BY−. In Experiment 1 participants had eight of each trial type presented in a random order, with order randomized for each participant subject to the constraint that no more than two trials of the same type could occur in sequence. The symbols and colors serving the cue functions A, B, X, and Y were selected at random for each participant from a set of 14 symbols and a set of 13 colors (e.g., Wingdings character 94 on a pink background). The background colors were allocated to role of informative cues (A and B) and the symbols allocated to the role of redundant cues (X and Y) in an approximately counterbalanced fashion so that 30 participants had colors in the A, B roles and foreground symbols in the X,Y roles; vice-versa for the remaining 31. In Experiment 2 participants had four trials of each type presented in one of five different orders, each order randomized subject to the constraint that no more than three trials of one type could occur in sequence. Four different symbols and three different colors were used. Allocation of colors and symbols to the role of informative (A and B) and redundant (X and Y) cues was approximately counterbalanced (*n* = 73 color predictive and *n* = 71 symbol predictive). In both experiments trials AX+ and AY+ were reinforced trials and were followed by the “win” outcome after participants made their judgements. Trials BX− and BY− were non-reinforced trials, and were followed by the “lose” outcome after participants made there judgements. Outcome feedback was in the form of onscreen text “win” and “lose” accompanied by distinctive auditory signals.

### Analyses

Analyses 1 used data from the 61 participants who took part in Experiment 1. Analyses 2 used data from the 144 participants who took part in Experiment 2. Both analyses each involved running four simulations. Simulations of the RW and the MECA models were both run twice against the data from each participant; once with the Stevens and once with the Fechner response mappings. The simulations were carried out in order to select optimized values for model parameters i.e., the simulations involved tuning the model parameters to produce responses matched as closely as possible to those actually made by the participant. The simulations were done using a computer program written in Java and using the Apache Commons Math implementation of Hansen's Covariance Matrix Adaptation Evolution Strategy (Hansen, 2006, 2012; Commons Math Developers, 2013). The Covariance Matrix Adaptation Evolution Strategy (CMAES) is a derivative-free multivariate optimization algorithm which was applied to an objective function that produced the sum of squared deviations (SSD), summed over all learning trials, between the participant's response and the model. The CMAES algorithm searched for best fitting parameters for the model such that the value of the objective function was minimized. Thus, the analyses yielded, for each participant and each model, a set of parameters and an SSD value as a measure of goodness of fit. The parameters involved included the α and β learning rate parameters for the RWM and MECAM (Equation A1 and Appendix Equation A5), the buffer weights for the MECAM (ω values, Appendix Equation A5), and the parameters used to control the mapping of association to response strength in the Fechner and Stevens models (Appendix Equations A3, A4). Further details of the simulation methods are given in the Appendix. Statistical tests were performed using the R statistics package (R Core Development Team, 2012).

## Results

The results are presented in four parts. First, the average learning curves from Experiment 1 and Experiment 2 are presented. Second, comparisons are made between the models using Stevens and Fechner response mappings. The Stevens response mapping produced better fits and, for brevity, some results are only presented graphically for the models with Stevens response mapping. Third, a comparison of the RW and MECA models is made. Finally, a comparison of the model parameters between Experiment 1 and Experiment 2 was made to determine their stability from one dataset to another. In the results that follow the SSD values found in the optimizations were converted to Root Mean Square (RMS) measures of goodness of fit. This was done to provide comparability between Experiment 1 and Experiment 2. This was necessary because Experiment 1 had 32 learning trials whereas there were only 16 trials in Experiment 2. Thus the SSD values for Experiment 1 were larger than those in Experiment 2. Because RMS error is the average error over all data points RMS magnitude is not directly affected by the length of the trial sequence.

### Average learning curves

**Figure 1:**
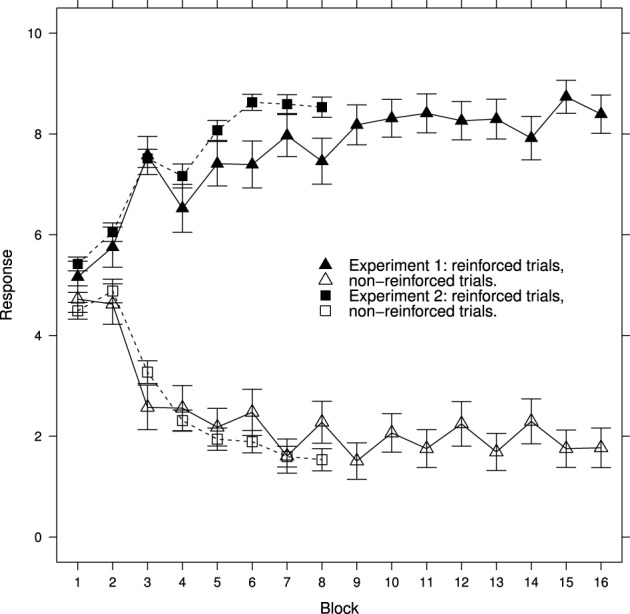
**Ratings for reinforced and non-reinforced trials mean ± standard error for Experiment 1 and Experiment 2**. See Results section for further details.

Figure 1 shows the average learning curves generated in Experiment 1 and 2. These curves show that learning has taken place, there are clear differences in responses to reinforced and non-reinforced cards after the second block of trials. However, for reasons described in the introduction, the learning functions for individual participants cannot be deduced from these averages. Furthermore, these average curves hide a great deal of detail at the level of individual learning curves. In order to address both of these issues each of the following figures shows an ordered selection of individual participant data.

### Comparison of Fechner and Stevens response mapping

**Figure 2:**
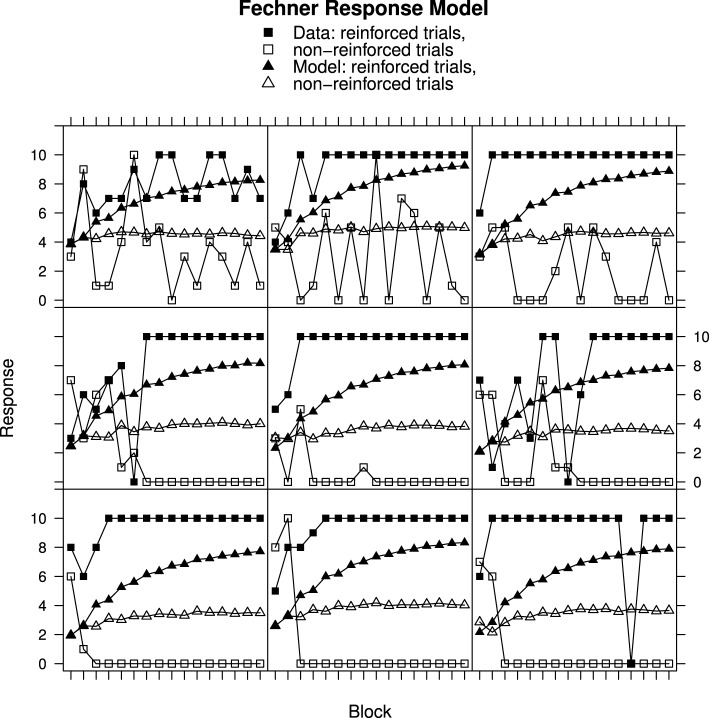
**Ratings and Rescorla–Wagner Model predictions for reinforced and non-reinforced trials using the Fechner Response Model in Experiment 1. Top row**, best fitting model samples. **Middle row**, intermediate fits. **Bottom row**, worst fitting model samples. See Results section for further details.

**Figure 3:**
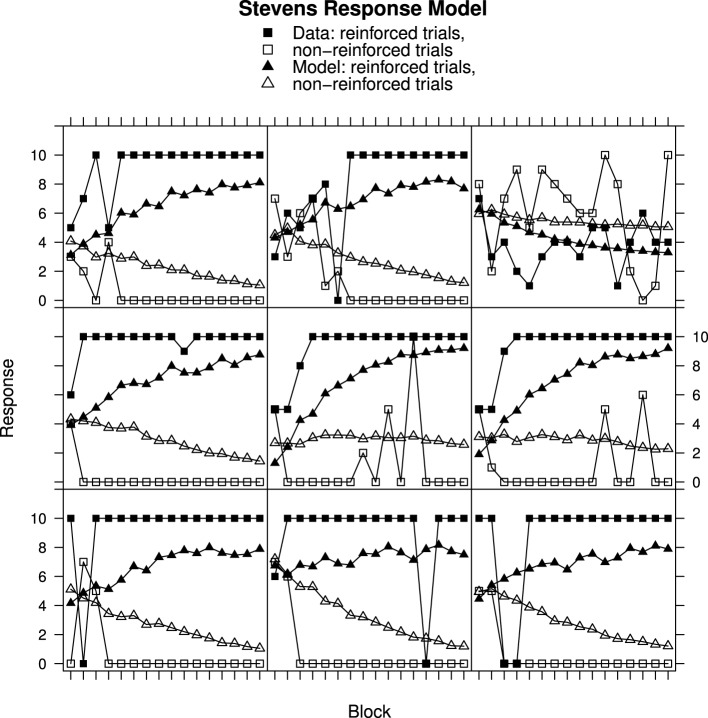
**Ratings and Rescorla–Wagner Model predictions for reinforced and non-reinforced trials using the Stevens Response Model in Experiment 1. Top row**, best fitting model samples. **Middle row**, intermediate fits. **Bottom row**, worst fitting model samples. See Results section for further details.

Figures 2, 3 show individual learning curves for samples of participants from Experiment 1 alongside model fits obtained for the Rescorla–Wagner Model equipped with the Fechner (Figure 2) and Stevens (Figure 3) response mapping models. Each figure contains nine graphics, each of which shows data for an individual participant and the associated best fitting model predictions. The data in the rows is selected to illustrate the variation in goodness of fit between model and data. The top rows represent best fits. They contain samples of participants from the lower tercile of the RMS error distributions. The middle rows contain samples of participants from the middle tercile of the RMS error distributions. The bottom rows represent worst fits. They contain samples of participants from the upper tercile of the RMS error distributions.

Figure 2 shows data from Experiment 1 plotted along with best fits from the Rescorla–Wagner Model using Fechner's equation for mapping associative strength to response. All of the participants featured in this figure have learned to respond appropriately to the reinforced and non-reinforced cards but in several cases (e.g., top-left panel) the participants' responses remain unstable, varying from trial-to-trial. The best fitting simulation responses mirror the overall discriminations made by the participants but do not capture the trial-to-trial variation in responding produced by the participants, nor the downward trend in response on the non-reinforced trials. It is notable that the worst model fits, in the bottom row, occur for participants who had quickly learned the discriminations. The poor fits occur because participant responses reach asymptote within the first few trials while the model responses slowly approach their asymptotes. This results in large discrepancies between data and model on the early trials. In contrast, in the top row, the fits are better because the participant responses asymptote more slowly. Analysis of Variance on these data produced a significant 3-way interaction [*F*_(30, 870)_ = 2.28, *p* < 0.001] of Block (1–16) × Reinforcement (non-reinforced “v” reinforced) × Group (Best, intermediate, and worst RMS fit) confirming that the development of the discrimination between non-reinforced and reinforced trials differed according to the model goodness of fit.

Turning to Figure 3, participant data from Experiment 1 is shown alongside Rescorla–Wagner Model best fits using Stevens' equation to map associative strength to response strength. All except one participant (top-right panel) in this sample has learned to respond appropriately. Once again the fits for the participants who learned very quickly are worse (bottom row) than for those who learned more slowly (top row) with ANOVA showing a significant interaction between Block, Reinforcement, and Group [*F*_(30, 870)_ = 2.30, *p* < 0.001]. In contrast to the Fechner based model, the model responses on the non-reinforced trials decline over trial blocks.

**Figure 4:**
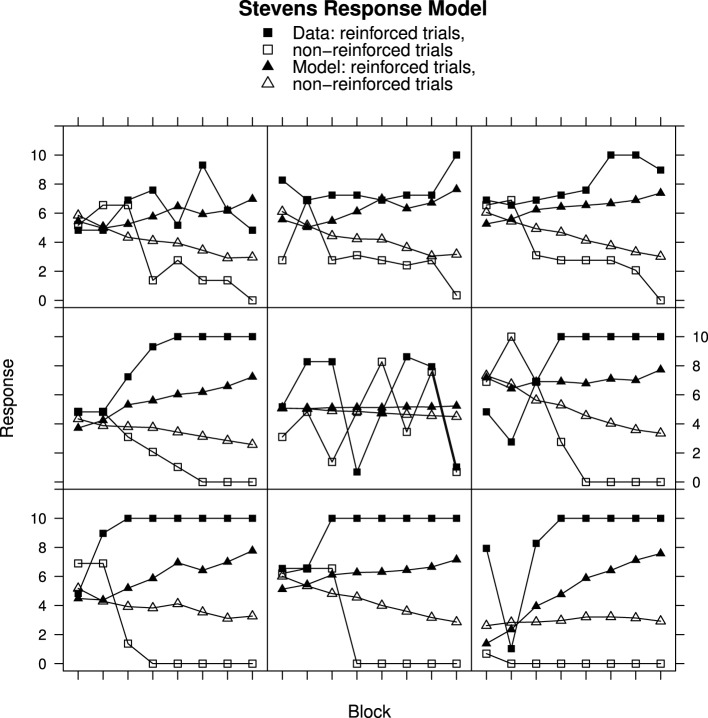
**Ratings and Rescorla–Wagner Model predictions for reinforced and non-reinforced trials using the Stevens Response Model in Experiment 2. Top row**, best fitting model samples. **Middle row**, intermediate fits. **Bottom row**, worst fitting model samples. See Results section for further details.

Student's *t*-tests on the RMS error showed that the mean RMS fit was significantly better for the Stevens Response Model than for the Fechner Response Model [*t*_(60)_ = 10.67, *p* < 0.001]. The mean RMS error values are given in Table 1. A very similar picture was obtained for the analysis of Experiment 2. For brevity a sample of participant and model data is presented for Experiment 2, only for the Stevens model, in Figure 4. ANOVA once again showed that the fit was related to the rate of discrimination [*F*_(14, 987)_ = 3.93, *p* < 0.001] and the Stevens response model also produced significantly better fits for the data of Experiment 2 than did the Fechner model [*t*_(143)_ = 19.63, *p* < 0.001].

**Table 1:** **Parameters values obtained in Analyses 1 and 2 and model goodness of fit values (RMS)**.

**Experiment**	**Model**	**α_ctx_**	**α_cue_**	**β_rt_**	**β_nrt_**	***k***	***a***	***c***	***sbw***	***ibw***	***sv***	**RMS**
**FECHNER RESPONSE MAPPING**												
1	RWM	0.03	0.24	0.24	0.20	9.10	–	1.71	–	–	0.04	3.24
		(0.008)	(0.006)	(0.005)	(0.011)	(0.236)	–	(0.111)	–	–	(0.010)	(0.078)
	MECAM	0.13	0.24	0.24	0.20	8.52	–	1.11	0.32	0.80	0.05	3.06
		(0.013)	(0.005)	(0.005)	(0.012)	(0.323)	–	(0.118)	(0.043)	(0.039)	(0.010)	(0.071)
2	RWM	0.04	0.24	0.24	0.22	9.40	–	2.25	–	–	0.04	2.91
		(0.006)	(0.004)	(0.002)	(0.005)	(0.107)	–	(0.087)	–	–	(0.005)	(0.057)
	MECAM	0.11	0.24	0.24	0.22	8.67	–	1.44	0.35	0.78	0.04	2.77
		(0.009)	(0.003)	(0.003)	(0.005)	(0.168)	–	(0.088)	(0.029)	(0.024)	(0.004)	(0.057)
**STEVENS RESPONSE MAPPING**												
1	RWM	0.18	0.23	0.23	0.20	8.91	2.25	–	–	–	0.12	2.82
		(0.013)	(0.008)	(0.006)	(0.012)	(0.290)	(0.107)	–	–	–	(0.007)	(0.068)
	MECAM	0.11	0.23	0.23	0.20	8.39	1.81	–	0.27	0.67	0.07	2.65
		(0.012)	(0.006)	(0.004)	(0.011)	(0.322)	(0.118)	–	(0.036)	(0.041)	(0.009)	(0.071)
2	RWM	0.18	0.24	0.24	0.23	9.26	2.53	–	–	–	0.12	2.60
		(0.008)	(0.004)	(0.002)	(0.005)	(0.144)	(0.067)	–	–	–	(0.004)	(0.055)
	MECAM	0.12	0.24	0.24	0.22	8.61	1.84	–	0.30	0.77	0.06	2.43
		(0.008)	(0.003)	(0.003)	(0.006)	(0.145)	(0.081)	–	(0.025)	(0.022)	(0.004)	(0.047)

*Means (standard error)*.

### Comparison of RWM and MECAM

**Figure 5:**
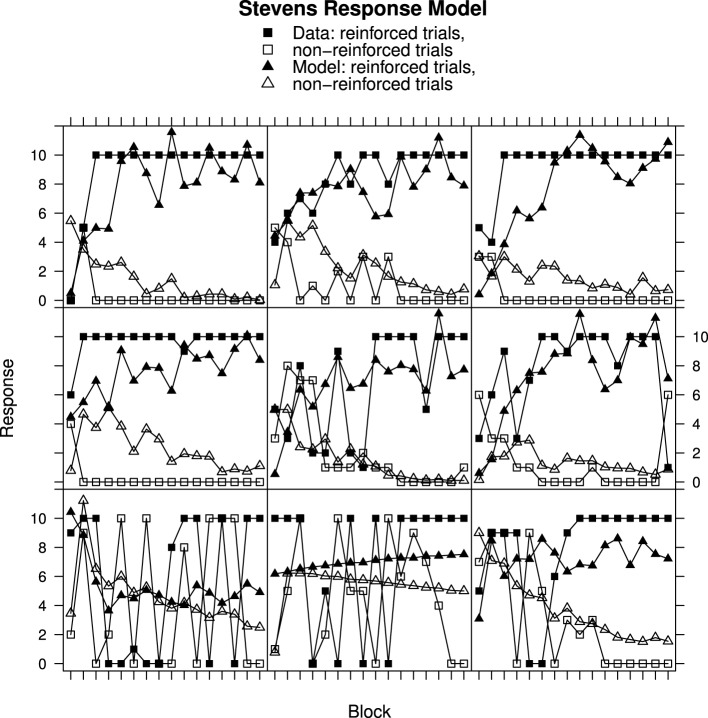
**Ratings and MECA Model predictions for reinforced and non-reinforced trials using the Stevens Response Model in Experiment 1. Top row**, best fitting model samples. **Middle row**, intermediate fits. **Bottom row**, worst fitting model samples. See Results section for further details.

**Figure 6:**
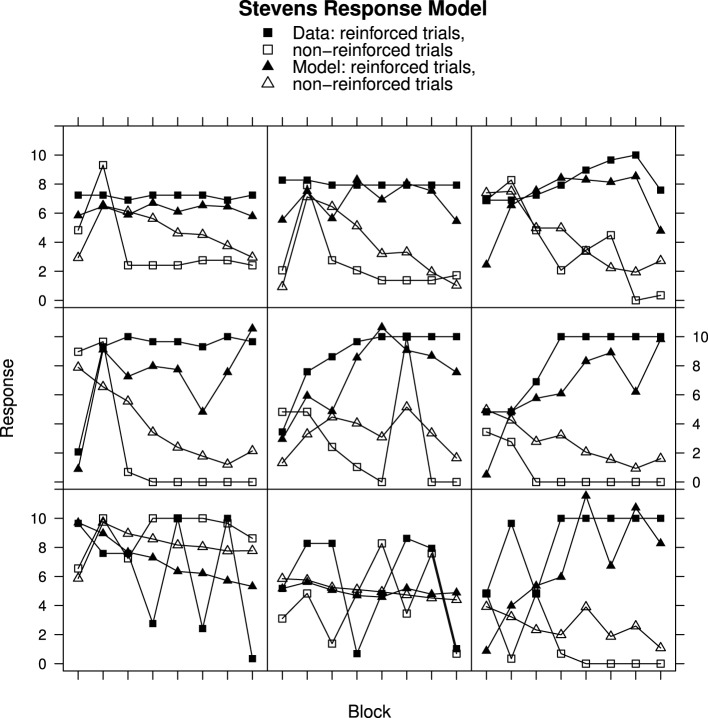
**Ratings and MECA Model predictions for reinforced and non-reinforced trials using the Stevens Response Model in Experiment 2. Top row**, best fitting model samples. **Middle row**, intermediate fits. **Bottom row**, worst fitting model samples. See Results section for further details.

Although the RWM captures the general trends in the data, particularly when using Stevens response mapping, consideration of the individual data in Figures 2–4 reveals that the fitted model does not accurately reproduce the participant responses. The MECA Model was developed as an alternative application of the Rescorla–Wagner principles. The aim was to determine whether or not these shortcomings of the Rescorla–Wagner Model might be rectified by using a more elaborate model of the stimulus environment. Figures 5, 6 show data from Experiments 1 and 2 together with best fits from the MECA Model using Stevens Response Model. In comparison with the Rescorla–Wagner Model fits (compare Figure 3 with 5 and Figure 4 with 6) the MECA Model produced good fits for the participants who learn quickly the correct responses, as well as good fits for the participants who learn more slowly. The three-way interaction of Block, Reinforcement, and Group was not significant in Experiment 1 [*F*_(30, 870)_ = 1.24] nor in Experiment 2 [*F*_(14, 987)_ = 1.50]. In addition to providing better fits overall the MECA Model also produced less stable responses from trial-to-trial and it is in that sense a better approximation to the responses produced by the participants. In many cases the trial-to-trial variation in the model predictions does not covary with the participant responses but in a number of cases there are striking correspondences (e.g., Figure 5 middle and middle-right panels). Student's *t*-tests on the RMS error showed that the mean RMS fit was significantly better for the MECA Model than for the RWM in Experiment 1 and in Experiment 2 [*t*_(60)_ = 5.68, *p* < 0.001 and *t*_(143)_ = 5.88, *p* < 0.001, respectively]. The RMS error values are given in Table 1.

**Figure 7:**
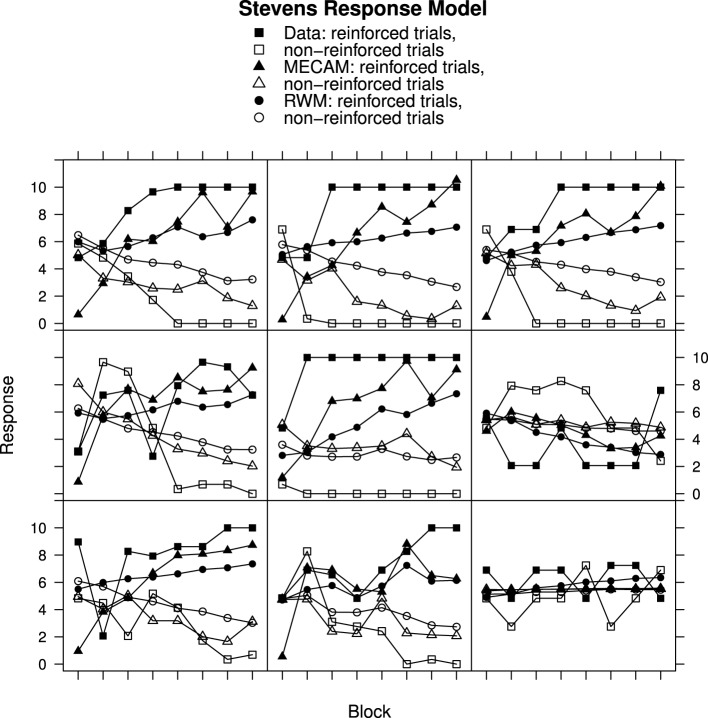
**Ratings, MECAM, and RWM predictions for reinforced and non-reinforced trials using the Stevens Response Model in Experiment 2. Top row**, greatest MECAM improvement samples. **Middle row**, intermediate MECAM improvements. **Bottom row**, least MECAM improvement samples. See Results section for further details.

For Experiment 1 there was an improvement in the RMS error value for the MECA Model over the RW Model in 43 out of 61 cases—70% of participants has better fits using the MECAM, the median improvement value was 0.11. In Experiment 2 the median improvement value of MECAM over the RWM was also 0.11 with the MECAM producing smaller RMS values in 89 out of 144 participants—62% had better MECAM fits than RWM fits. Figure 7, gives direct comparisons of the fits of the MECAM and RWM to a selection of individual participants from Experiment 2. Each panel shows data from a single participant and the best fitting RWM and MECAM responses to facilitate comparison of the models. The rows in Figure 7 are arranged to show tercile samples for participants varying according to the improvement in fit that the MECAM provided over the RWM. Participants were ranked according to the difference in RMS values between the model fits (RWM minus MECAM). A positive value on this difference score indicates that the MECAM model had a better fit than the RWM. In Figure 7 the top row provides a sample of participants from the upper tercile of the improvement distribution (most improvement), the middle row a sample from the middle tercile, and the bottom row a sample from the lower tercile (least improvement). From left to right the RMS improvements in the top row were 0.46, 0.76, and 0.74; for the middle row they were 0.28, 0.32, and −0.01; and for the bottom row they were −0.26, −0.04, and −0.05.

### Comparison of parameters from experiment 1 and experiment 2

Multivariate Analysis of Variance (MANOVA) was used to compare Experiment 1 and Experiment 2 to assess whether or not the fitted model parameters differed for the two datasets. The parameter values for Experiment 1 and Experiment 2 did not differ in three out of the four cases. The parameters were the same in both datasets for the MECA Model with Fechner response mapping, and for the MECAM and RW Models with Stevens response mapping [approximate Fs *F*_(9, 195)_ = 1.61, *F*_(9.195)_ = 0.84, and *F*_(7, 197)_ = 1.42, respectively]. MANOVA did show a difference between experiments when the RWM with Fechner response mapping was considered [approximate *F*_(7, 197)_ = 9.38, *p* < 0.001]. Follow-up *t*-tests using Welch's correction produced significant differences only for the response mapping parameter *c*. Lower values of *c* were found in Experiment 1 than in Experiment 2 [*t*_(135)_ = 3.81, *p* < 0.001].

## Discussion

Two principle findings emerged. First, in this model fitting exercise, better results were obtained by using a mapping between associative strength and response strength based on Stevens' Power Law than by using a mapping based on Fechner's Law. The average model predictions using the RWM and Stevens response mapping differed from the participant data by 2.82 (Experiment 1) and 2.60 (Experiment 2) units on an 11 point response scale. In comparison the same figures for the Fechner response mapping were 3.24 and 2.91 (see RMS values in Table 1). Second, although the RWM captured general trends in the individual data, the fits were poor and significant improvements were obtained using the MECAM. Using Stevens response mapping the average MECAM predictions differed by 2.65 and 2.43 units from the participant data (Experiment 1 and Experiment 2, respectively). This latter result supports the main hypothesis of this work, that participant responses on trial n are influenced by the predictive value of the memorial representations of stimuli from the previous trial. Since the sequences in these experiments were generated randomly it is argued that the predictive contributions of trial *n* − 1 memory stimuli serve to add noise to the observed responses. Because these stimuli are unlikely to remain predictive for long sequences of trials they will tend to lose their influence toward the end of the trial sequence.

The introduction began with a statement of the theoretical significance of the form of the learning curve. Although analysis of learning curves appeared to offer a route for theory advance, the promise of ruling in or out one of two major classes of learning curve (power or exponential) has not been fulfilled. Several factors have contributed to the difficulties including using multicomponent tasks and problems with averaging (e.g., Mazur and Hastie, 1978; Heathcote et al., 2000). However, even if it is not possible to clearly determine whether or not learning curves are best characterized by power functions or by exponential functions, this does not exhaust the possibilities for theoretical analysis offered by a study of learning curves. Individual learning curve data are highly variable and idiosyncratic, and we do not yet have an accurate theoretical model of this variability. Some have argued for alternatives to associative models to understand these data (e.g., Nosofsky et al., 1994; Gallistel et al., 2004). Here it is argued here that an associative model of individual learning curves is worthy of further exploration but that such a model will require a more realistic approach to characterizing the environment of the learner. The current MECA Model is one example of such a strategy and one of its core assumptions is that “non-local” features play a part in this environment. A second core assumption in the MECAM is that an adequate description of the stimulus environment will require recognition of interactions between elemental stimuli. Both of these core assumptions were examined in the current investigation and will be discussed below.

This is not the first time that it has been suggested that there are non-local influences on behavior. The Perruchet effect mentioned in the introduction is another example (Perruchet et al., 2006) and there have been related suggestions in studies of sequence learning effects. Theoretical analyses of non-local influences have been explored previously in the framework of Simple Recurrent Networks (SRNs) as well as in memory buffer frameworks similar to that used in the MECAM. In the original SRN model (Elman, 1990) a three-layer neural network was used with the activations of the hidden-layer fed-back to form part of the input pattern for the current trial. This SRN was introduced as an alternative to memory buffer models of sequence learning in which the inputs of previous trials were simply repeated on the current trial. The SRN approach to sequence learning has acquired prominence but memory buffer models still appear to have some utility. Kuhn and Dienes found that a memory buffer model of learning better approximated human learning than did an SRN model (Kuhn and Dienes, 2008). Of course there are many ways in which a memory buffer model could operate and the challenge now is to develop an optimal approach. In their buffer model Kuhn and Dienes used the previous four trials and did not include any configural cue representations. The MECA Model presented here adopted a memory buffer approach using just the previous trial and included representations of configural cues. The MECAM's implementation of both of these ideas requires further examination and development.

Use of two trials *t* and *t*_*n* − 1_ is only an approximation to modeling the continuous time-based nature of experience. However, as argued in the introduction and as demonstrated empirically, inclusion of trial *t*_*n* − 1_ results in qualitative and quantitative improvements in modeling of simple learning as compared to the same model using trial *t* alone. Further investigation of this approach could be carried out by using additional buffers to determine an optimal number but a more principled approach to further development of the MECAM is preferred. In MECAM the primary buffer is a focal memory store containing the events of the current trial and the secondary buffer contains a remembered version of the previous trial. The interaction buffer is a configural product of the elements in the primary and secondary buffers. MECAM currently represents time by trial-based discrete changes in the contents of these primary and secondary buffers the consequence of which is that only the current and previous trial events can be learned about. One way to allow the possibility of events from trial *t*_*n* − *x*_ to play a part in MECAM's learning would be to include a model of decay and movement of the elements between the primary and secondary buffers. This would allow the buffers to contain a more heterogeneous representation of previous trials, for example the bulk of the secondary buffer could be occupied with memories of trial *t*_*n* − 1_ with progressively smaller components representing trial *t*_*n* − 2_, *t*_*n* − 3_ etc. Discussion of the model of buffer behavior is beyond the scope of this article but is emphasized that even a crude operationalisation of this aspect of MECAM is an improvement on modeling solely with trial *t* alone.

The inclusion of configural cues in the MECAM may seem questionable because there is no requirement that participants use configural cues to respond appropriately in the tasks used. Whilst some studies have shown that the weight attached to configural cues can be increased by experience (e.g., Melchers et al., 2008) there is also data to indicate that configural processes operate by default, rather than simply coming into play as necessary (e.g., Shanks et al., 1998). Thus, the simplifying assumption to exclude configural cues seems no more justified than assuming participants would only attend to the current trial. Indeed, part of the rationale for MECAM was to include aspects of the stimulus environment that are, strictly speaking, redundant for the solution of the problem at hand. The MECAM assumes that participants are responding to something when “noisy responses” occur and takes into account *measurable* components of environmental structure which previous studies have shown, in other contexts, to be important in controlling responding. It should be noted here though that the modeling exercise did not include specific comparisons of the standard RWM with and without configural cues. The primary focus was on the comparison of two models, both containing configural cues, with one model only representing the current trial (the RWM) and the other model representing the current and previous trial (the MECAM). Nevertheless we can assert that configural cues are important by looking at the optimized values of the interaction buffer weight in Table 1. In all fitted models this weight is substantially greater than zero and since the interaction buffer contains only configural cues this result supports their inclusion in modeling. The result for the secondary buffer is not as clear because this buffer contains a mixture of configural and elemental stimulus representations.

Thus, the MECAM principle of including an extended description of the stimulus environment, in terms of both trial history and stimulus interactions, is a reasonable way to reconcile an associative model such as the RWM with the learning curve data but the extent to which MECAM can be refined remains to be determined; MECAM as it stands is far from a complete account. The current work has provided some proof-of-concept for two major principles and future work is needed for refinement. A suggestion for a more flexible model of buffer behavior has already been mentioned and there is also a need to explore of different types of configural cue model apart from the pairwise stimulus unique-cue model used in this version of MECAM (e.g., Brandon et al., 2000).

Further developments of MECAM are justified on the basis of the statistically significant, and visible improvements, to the modeling of individual learning curves that were obtained in the current work. However, one criticism that could be leveled at the MECAM is that the gains are small and that the model is excessively complex. Examination of the RMS error values in Table 1 provides a metric against which to assess the size of the gains. In Experiment 1, for the Stevens response mapping, the RMS error for the MECAM was 6% less than for the RWM; in Experiment 2 the RMS error reduction was 6.5%. In these simulations MECAM was implemented with nine free parameters, a considerable increase from the RWM implied by Equation (1), which appears to include only two free parameters, α and β. It is true that the RWM is a simple model but in reality most applications of the model actually use more than these two explicitly declared free parameters. It is common practise to allow different values of α for different cue types (e.g., context cues and configural cues may have lower values) and different β values for reinforced and non-reinforced trials (e.g., Mondragon et al., 2013). If the model is intended to make quantitative rather than just qualitative predictions then inclusion of a rule to map associative strength to response strength necessarily introduces additional parameters. In the current simulations the RWM was implemented with seven free parameters so the MECAM effectively included two additional free parameters, the weights for the primary and secondary buffers *sbw* and *ibw*. It is well beyond the scope of the current paper to provide a detailed discussion of whether or not the observed gains are worth the cost of the additional parameters but two points are worthy of note. First, model complexity is not determined solely by the number of free parameters in the model (Grünwald, 2005). In fact, compared with some leading learning models (for a recent review see Wills and Pothos, 2012) the MECAM remains algorithmically simple, using the standard RWM learning rule. The aim of MECAM was to retain algorithmic simplicity and find a suitable account of the observed individual behavioral complexity in terms of the observable environmental events experienced by individual participants. Second, the model parameters were stable in two different datasets, this replication gives some assurance of the model generality.

The current test of MECAM was focussed on its ability to generate better fits to learning curve data but there are a number of other model specific predictions that would valuable to establish the psychological validity of the concepts in MECAM. For example, because MECAM predicts an influence of the previous trial on responding to the current trial then it follows that an alternating sequence of A− and B+ trials would be learned more quickly than when A− and B+ trials were presented in a random order. Furthermore MECAM would predict considerable responding, following the alternating sequence, on the second trial of a test consisting of the sequence B− followed by T, where T is a novel test stimulus. After a randomly ordered sequence of A− and B+ trials a test consisting of B− followed by T should elicit relatively little responding. The MECAM would also give rise to the prediction that participants with better short-term memories^1^ would likely have increased salience of events on trial *t*_*n* − 1_ and thus respond differentially to a manipulation involving trial orderings. This type of test, involving model specific predictions, will ultimately be required to justify the additional complexity of the MECAM. It is clear though that we are currently in a rather uncomfortable position because models such as the RWM are unable to provide accurate quantitative approximations to the observed learning curves—a fact which is a significant shortcoming in the field of learning research.

In summary, a simple associative model such as the RWM gives only a poor approximation to individual learning curve data. It is not appropriate to rely on analysis of average curves to resolve this problem but a viable theory of learning must still be able to provide an accurate model of the individual data. The MECAM is a development of the RWM which attempts to model the complex responses that make up individual learning curves. The MECAM assumes that participant responses are subject to non-local influences (e.g., cues present on previous trial) and, because these cues are typically not predictive for long trial sequences, the influence of these cues adds noise to the observed learning curves. The improvements made by the MECA Model over the RWM suggests that this assumption is reasonable and the cue-structures defined in the current investigation are offered as an initial approximation subject to further investigation.

### Conflict of interest statement

The authors declare that the research was conducted in the absence of any commercial or financial relationships that could be construed as a potential conflict of interest.

## Footnotes

^1^I am grateful to a reviewer of this paper for this suggestion.

## Appendix

### Simulations

Details of the simulations of the RWM and the MECAM are provided below. Table A1 provides a summary of the model parameters. Table A2 illustrates the operation of the MECAM memory buffers.

**Table A1:** **Parameters used, and optimization boundary values, in RWM and MECAM simulations**.

**Parameter**	**Description**	**Lower bound**	**Upper bound**	**MECAM**	**RWM**
α_ctx_	α value for context	0.01	0.25	✓	✓
α_cue_	α value for cues	0.01	0.25	✓	✓
β_rt_	β for reinforced trials	0.01	0.25	✓	✓
β_nrt_	β scaling for non-reinforced trials	0.01	0.25	✓	✓
*k*	Associative strength to response mapping	0.01	10	✓	✓
*a*	Associative strength to response mapping	0.01	3	✓	✓
*c*	Associative strength to response mapping	0.01	5	✓	✓
*sbw*	Weight for secondary buffer stimuli	0	1	✓	×
*ibw*	Weight for interaction buffer stimuli	0	1	✓	×
*sv*	Initial associative strength	0.01	0.25	✓	✓

**Table A2:** **State of buffers on three successive trials; AB+, ABC−, and BC−**.

	**Trial 1**		**Trial 2**		**Trial 3**	
	**Element cues**	**Configural cues**	**Element cues**	**Configural cues**	**Element cues**	**Configural cues**
Primary buffer	A	ab	A	ab	B	bc
	B		B	ac	C	
			C	bc		
Secondary buffer			A_*t* − 1_	a_*t* − 1_b_*t* − 1_	A_*t* − 1_	a_*t* − 1_b_*t* − 1_
			B_*t* − 1_		B_*t* − 1_	a_*t* − 1_c_*t* − 1_
			O_*t* − 1_		C_*t* − 1_	b_*t* − 1_c_*t* − 1_
Interaction buffer				aa_*t* − 1_		ba_*t* − 1_
				ab_*t* − 1_		bb_*t* − 1_
				ao_*t* − 1_		bb_*t* − 1_
				ba_*t* − 1_		ca_*t* − 1_
				bb_*t* − 1_		cb_*t* − 1_
				bo_*t* − 1_		cc_*t* − 1_
				ca_*t* − 1_		
				cb_*t* − 1_		
				co_*t* − 1_		

*Element cue O_t − 1_ on trial 2 is the memory of the outcome that occurred on trial 1. See text above (page 16)*.

#### RWM simulations

The RWM simulations used the standard Rescorla–Wagner equation (Rescorla and Wagner, 1972) for updating associative strength for each stimulus present on a trial *t*, namely:

(A1) $$V_{i,t+1}=V_{i,t}+\alpha_{i}\beta (\lambda_{t}-\sum_{i=1}^{n}V_{i,t})$$

In Equation (A1) the subscript *t* indexes the trial number and there are *n* stimuli present on a trial, indexed by subscript *i*. The update on associative strength *V*, is a product of α, β, and the parenthesized *error term*. λ is set to 0 for non-reinforced trials and 1 for reinforced trials. Implementation of Equation (A1) was carried out with the representation of each trial encoded to include the context, the explicit experimenter defined cues, and configural cues. For example, on an AX trial, six stimuli would be assumed to be present—C, A, X, ca, cx, and ax, where C is the experimental context (which was constant in all trials in the current simulations), A and X are the experimenter provided cues (foreground symbol and background color of the cards), and ca, cx, and ax are configural cues arising from pairwise interactions between stimulus elements C, A, and X.

The CMAES optimizing algorithm adjusted the α and β values used in Equation (A1). The α values for the configural cues were set to the average α value of the configuration elements divided by the number of elements represented (Equation A2). This scaling was chosen rather than selecting an arbitrary value on the basis that it provides a link between the salience of the elements and the configural cues, and reduces the salience of configural cues relative to element cues. Separate α values for the context and cues were selected by the optimizer, parameters α_ctx_ and α_cue_. On reinforced trials β was set to the parameter β_rt_ and on non-reinforced trials this was scaled by multiplication with parameter β_nrt_. The optimizer also selected the initial associative strength for all cues at the start of each simulation, parameter *sv*, and the parameters to control the mapping of associative strength to response strength. Optimization of *sv* was provided as an alternative to setting initial strength to zero or to a random value. Two models were used for response mapping, both of these use two parameters. For the mapping based on Stevens' Power Law the model response was given by Equation (A3) and for Fechner's Law the model response was given by Equation (A4). The optimizations minimized the sum of squared deviations between the model and participant responses, summing over all trials. Constraints were applied to the parameters, for RWM and MECAM simulations, as shown in Table A1 because simulations became unstable in some cases without constraints.

(A2) $$\frac{\Sigma_{\alpha }}{n^{2}}$$

(A3) $$k{\left[\sum_{i=1}^{n}V_{i,t}\right]}^{a}$$

(A4) $$k\ln\left[\sum_{i=1}^{n}V_{i,t}+1\right]+c$$

#### MECAM simulations

The MECAM simulations used a modification of Equation (A1):

(A5) $$V_{i,t+1}=V_{i,t}+\omega \alpha_{i}\beta \left(\lambda_{n}-\sum_{i=1}^{n}V_{i,t}\right)$$

In Equation (A5) an additional parameter ω is used to adjust the update to the associative strength of each cue that is present on a trial. In the MECAM the stimulus environment is assumed to consist of stimulus representations in three buffers, a *primary buffer*, a *secondary buffer*, and an *interaction buffer*. The value of ω is determined for each cue according to the buffer in which the cue is defined. The primary buffer holds representations of the stimuli present on the current trial, as specified in the implementation of the RWM described above. ω for primary buffer stimuli is set at 1. The secondary buffer holds representations of the stimuli that were present on the previous trial. ω for secondary buffer stimuli was set at the value adjusted by the optimizer, the parameter secondary buffer weight (*sbw*) is shown in Table A1. The primary and secondary buffers both hold elemental representations of stimuli and pairwise configural cue representations of the elemental cues as shown in Table A2. In Table A2 stimuli from the previous trial are subscripted *t* − 1 and configural cues are in lower case. For example *A*_*t* − 1_ and *a*_*t* − 1_ *b*_*t* − 1_ represent memories from the previous trial. *A*_*t* − 1_ is the memory of element A and *a*_*t* − 1_*b*_*t* − 1_ is the memory of the configural cue for the co-occurrence of A and B. Note that the configural cues in the secondary buffer are remembered versions of those were created from pairwise combinations of the *stimuli* that were presented on the previous trial, they have not been created *de novo*. The interaction buffer, on the other hand, holds only configural cue representations. These representations are created from combinations of the element cues present in the primary and secondary buffers. For example *aa*_*t* − 1_ is the configural cue for the co-occurrence of element A on the current trial and the memory of A from the previous trial. Note that no new configural representations that appear in the interaction buffer are created entirely from remembered elements. Thus, in the tabulated example on trial 2, we obtain configural cues such as *aa*_*t* − 1_ because these consist of a current and a remembered element. However, we do not get cues such as *a*_*t* − 1_*o*_*t* − 1_ because this would involve two remembered elements. Configural cues involving two remembered elements only occur in the secondary buffer as remembered versions of configurations from the previous trial (e.g., *a*_*t* − 1_*b*_*t* − 1_).

ω for interaction buffer stimuli was set at the value adjusted by the optimizer, the parameter interaction buffer weight (*ibw*) is shown in Table A1. For further illustration of how the stimulus environment is represent in the MECAM refer to Table A2 which shows the state of the MECA buffers state in a series of three successive trials. Cues A and B are present on the first trial, and the outcome occurs; cues A, B, and C are present on the second, non-reinforced, trial; cues B and C are present on the third, non-reinforced trial.

## References

[B1] Atkinson R. Estes W. (1963). Stimulus Sampling Theory, chapter 10. Vol. 3 New York, NY: Wiley, 121–268

[B2] Brandon S. Vogel E. Wagner A. (2000). A componential view of configural cues in generalization and discrimination in pavlovian conditioning. Behav. Brain Res. 110, 67–72 10.1016/S0166-4328(99)00185-0 10802304

[B3] Chapman G. Robbins S. (1990). Cue interaction in human contingency judgement. Mem. Cogn. 18, 537–545 10.3758/BF03198486 2233266

[B4] Commons Math Developers. (2013). Apache Commons Math, Release 3.2. Available online at: http://commons.apache.org/math/download_math.cgi

[B5] Dickinson A. Shanks D. Evenden J. (1984). Judgment of act-outcome contingency - the role of selective attribution. Q. J. Exp. Psychol. Sect. A Hum. Exp. Psychol. 36, 29–50 10.1080/14640748408401502

[B6] Elman J. L. (1990). Finding structure in time. Cogn. Sci. 14, 179–211 10.1207/s15516709cog1402_1

[B7] Estes W. (1950). Toward a statistical theory of learning. Psychol. Rev. 57, 94–107 10.1037/h0058559

[B8] Estes W. (2002). Traps in the route to models of memory and decision. Psychonom. Bull. Rev. 9, 3–25 10.3758/BF03196254 12026952

[B9] Gallistel C. Fairhurst S. Balsam P. (2004). The learning curve: implications of a quantitative analysis. Proc. Natl. Acad. Sci. U.S.A. 101, 13124–13131 10.1073/pnas.0404965101 15331782 PMC516535

[B10] Gallistel C. Gibbon J. (2002). The Symbolic Foundations of Conditioned Behavior. Mahwah, NJ: Lawrence Erlbaum Associates Publishers

[B11] Grünwald P. (2005). Minimum Description Length Tutorial. Neural Information Processing Series (MIT Press, Grünwald, Peter, Centrum voor Wiskunde en Informatica Kruislaan 413, 1098 SJ, Amsterdam, Netherlands, pdg@cwi.nl), 23–79

[B12] Hansen N. (2006). The CMA evolution strategy: a comparing review. Towards New Evol. Comput. 192, 75–102 10.1007/3-540-32494-1_4

[B13] Hansen N. (2012). The CMA evolution strategy. Availble online at: https://www.lri.fr/~hansen/cmaesintro.html Retrieved from 2 July 2013.

[B14] Heathcote A. Brown S. Mewhort D. J. K. (2000). The power law repealed: the case for an exponential law of practice. Psychon. Bull. Rev. 7, 185–207 10.3758/BF03212979 10909131

[B15] Jones F. W. McLaren I. P. L. (2009). Human sequence learning under incidental and intentional conditions. J. Exp. Psychol. Anim. Behav. Process. 35, 538–553 10.1037/a0015661 19839706

[B16] Köhler W. (1925). The Mentality of Apes. New York, NY: Harcourt, Brace & Co

[B17] Krueger L. E. (1989). Reconciling fechner and stevens: toward a unified psychophysical law. Behav. Brain Sci. 12, 251–267 10.1017/S0140525X0004855X

[B18] Kuhn G. Dienes Z. (2008). Learning non-local dependencies. Cognition 106, 184–206 10.1016/j.cognition.2007.01.003 17343839

[B19] Lachnit H. (1988). Convergent validation of information-processing constructs with pavlovian methodology. J. Exp. Psychol. Hum. Percept. Perform. 14, 143–152 10.1037/0096-1523.14.1.143 2964503

[B20] Mazur J. E. Hastie R. (1978). Learning as accumulation - re-examination of learning curve. Psychol. Bull. 85, 1256–1274 10.1037/0033-2909.85.6.1256 734012

[B21] Melchers K. Lachnit H. Shanks D. (2008). Stimulus coding in human associative learning: flexible representation of parts and wholes. Behav. Process. 77, 413–427 10.1016/j.beproc.2007.09.013 18031954

[B22] Miller R. Barnet R. Grahame N. (1995). Assessment of the Rescorla–Wagner model. Psychol. Bull. 117, 363–386 10.1037/0033-2909.117.3.363 7777644

[B23] Mondragon E. Alonso E. Fernandez A. Gray J. (2013). An extension of the Rescorla and Wagner Simulator for context conditioning. Comput. Methods Program. Biomed. 110, 226–230 10.1016/j.cmpb.2013.01.016 23453075

[B24] Myung I. J. Kim C. Pitt M. A. (2000). Toward an explanation of the power law artifact: insights from response surface analysis. Mem. Cogn. 28, 832–840 10.3758/BF03198418 10983457

[B25] Newell A. Rosenbloom P. S. (1981). Mechanisms of Skill Acquisition and the Law of Practice. Hillsdale, NJ: Erlbaum, 1–55

[B26] Nosofsky R. Palmeri T. McKinley S. (1994). Rule-plus-exception model of classification learning. Psychol. Rev. 101, 53–79 10.1037/0033-295X.101.1.53 8121960

[B27] Pearce J. (1994). Similarity and discrimination - a selective review and a connectionist model. Psychol. Rev. 101, 587–607 10.1037/0033-295X.101.4.587 7984708

[B28] Perruchet P. Cleeremans A. Destrebecqz A. (2006). Dissociating the effects of automatic activation and explicit expectancy on reaction times in a simple associative learning task. J. Exp. Psychol. Learn. Mem. Cogn. 32, 955–965 10.1037/0278-7393.32.5.955 16938039

[B29] R Core Development Team (2012). R: a language and environment for statistical computing. Available onlline at: http://www.R-project.org/

[B30] Rescorla R. (1973). Evidence for a “unique stimulus” account of configural conditioning. J. Comp. Physiol. Psychol. 85, 331–338 10.1037/h0035046

[B31] Rescorla R. (2001). Are associative changes in acquisition and extinction negatively accelerated? J. Exp. Psychol. Anim. Behav. Process. 27, 307–315 10.1037/0097-7403.27.4.307 11676082

[B32] Rescorla R. Wagner A. (1972). A Theory of Pavlovian Conditioning: Variations in the Effectiveness of Reinforcement and Non-reinforcement. New York, NY: Appleton Century Crofts, 64–69

[B33] Rock I. (1957). The role of repetition in associative learning. Am. J. Psychol. 70, 186–193 10.2307/1419320 13424758

[B34] Roediger Arnold K. M. (2012). The one-trial learning controversy and its aftermath: remembering rock (1957). Am. J. Psychol. 125, 127–143 10.5406/amerjpsyc.125.2.0127 22774677 PMC4989509

[B35] Shanks D. Charles D. Darby R. Azmi A. (1998). Configural processes in human associative learning. J. Exp. Psychol. Learn. Mem. Cogn. 24, 1353–1378 10.1037/0278-7393.24.6.1353 9556907

[B36] Sidman M. (1952). A note on functional relations obtained from group data. Psychol. Bull. 49, 263–269 10.1037/h0063643 14930162

[B37] Sutton R. Barto A. (1981). Toward a modern theory of adaptive networks - expectation and prediction. Psychol. Rev. 88, 135–170 10.1037/0033-295X.88.2.135 7291377

[B38] Thorwart A. Glautier S. Lachnit H. (2010). Convergent results in eyeblink conditioning and contingency learning in humans: addition of a common cue does not affect feature-negative discriminations. Biol. Psychol. 85, 207–212 10.1016/j.biopsycho.2010.07.002 20638441

[B39] Wills A. J. Pothos E. M. (2012). On the adequacy of current empirical evaluations of formal models of categorization. Psychol. Bull. 138, 102–125 10.1037/a0025715 22061692

